# Hypertension screening via awake–sleep differences in photoplethysmogram signals

**DOI:** 10.1093/ehjdh/ztag131

**Published:** 2026-08-13

**Authors:** Jingyuan Hong, Manasi Nandi, Yali Zheng, Jordi Alastruey

**Affiliations:** School of Imaging Sciences and Biomedical Engineering, King's College London, St Thomas' Hospital, London SE1 7EH, UK; School of Cancer and Pharmaceutical Science, King’s College London, London, UK; College of Health Science and Environmental Engineering, Shenzhen Technology University, Shenzhen, China; School of Imaging Sciences and Biomedical Engineering, King's College London, St Thomas' Hospital, London SE1 7EH, UK

**Keywords:** Hypertension, Photoplethysmogram, Ambulatory monitoring, Machine learning

## Abstract

**Aims:**

Hypertension is a major risk factor for cardiovascular diseases. This study proposes a novel hypertension community-based screening framework based on intra-subject awake–sleep differences in photoplethysmography (PPG) indices, using machine learning. We hypothesized that normotensive individuals exhibit greater PPG variation between awake and sleep states than unmanaged hypertensive individuals.

**Methods and results:**

The Aurora-BP dataset (*n* = 180; 138 normotensive, 42 hypertensive) was used for model development, with 18 subjects reserved for internal testing. External validation was performed using the independent CUHK-BP dataset (*n* = 26; 11 normotensive, 15 hypertensive). Twenty PPG-based indices were extracted, and subject-level *P*-values from Mann–Whitney *U*-tests comparing awake and sleep periods were used as model features. Discretized *P*-values served as inputs for four machine learning models. The support vector machine (SVM) achieved the highest performance, with 81.1 ± 8.4% accuracy and 82.8 ± 8.1% F1-score on the internal test set using all indices. On the external test set, the SVM using only temporal indices achieved 84.6% accuracy and 86.7% F1 score. Temporal indices, especially those linked to the dicrotic notch, showed strong generalizability across datasets.

**Conclusion:**

The study demonstrates the feasibility of awake–sleep PPG analysis for hypertension screening, highlighting the potential of wearable PPG devices for ambulatory monitoring.

## Graphical Abstract

**Graphical Abstract:**
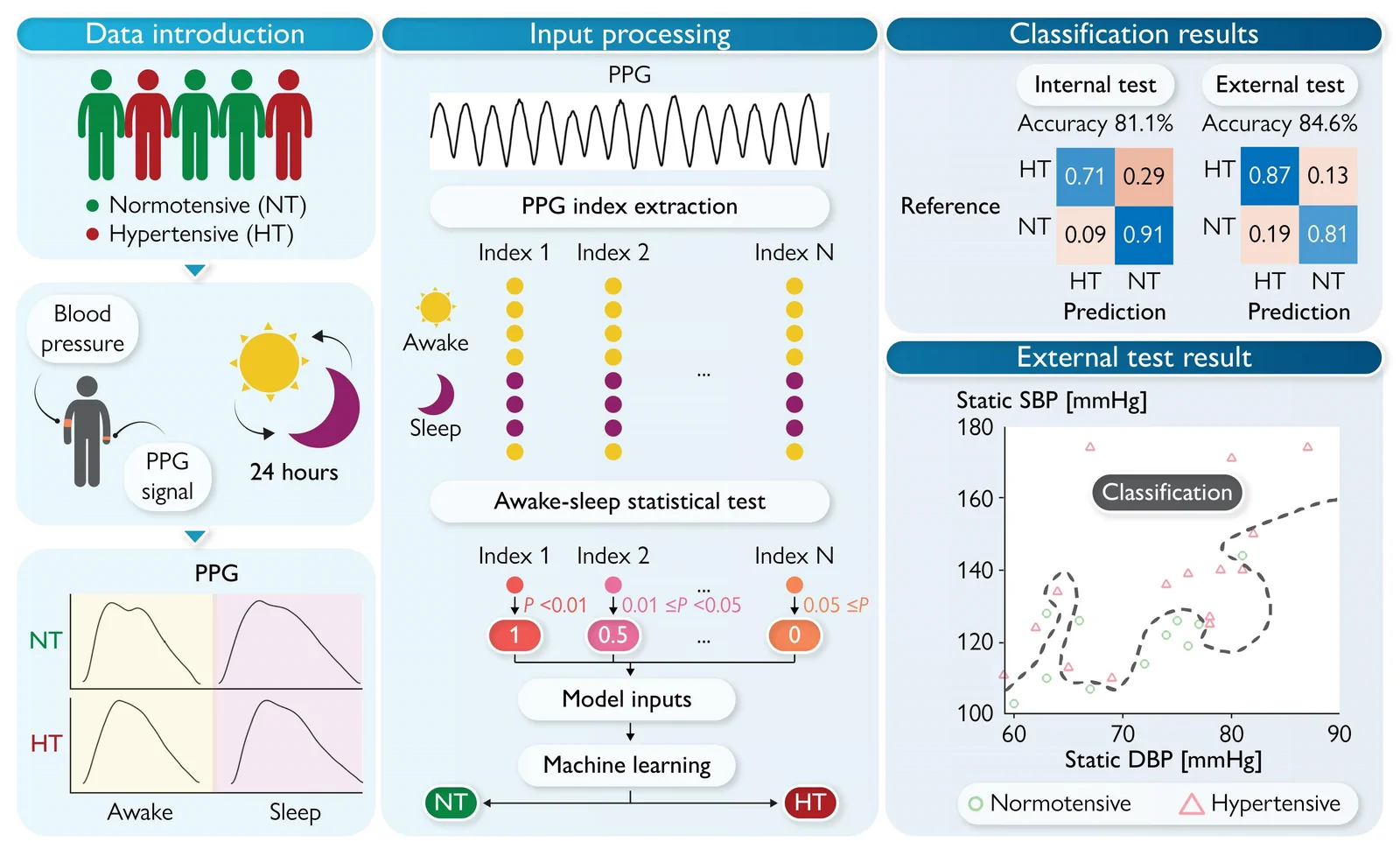

Novelty and relevance **What is new?**

- This study introduces a novel machine learning–based hypertension screening method using intra-subject awake–sleep differences in ambulatory photoplethysmogram (PPG) signals, validated on both internal and external datasets with a novel *P*-value–based feature selection strategy.

**What are the clinical implications?**

- The proposed approach enables non-invasive, cuff-free hypertension risk-alerts using wearable PPG data, offering improved generalizability and real-world applicability over traditional models.
- The ability to detect altered nocturnal cardiovascular patterns from PPG may support earlier identification of unmanaged hypertension and promote more accessible long-term monitoring.

## Introduction

Hypertension is a leading modifiable risk factor for cardiovascular mortality.^1^ It is typically diagnosed using upper-arm cuff devices that estimate brachial blood pressure (BP) via oscillometry.^2^ Diagnosis strategies range from repeated office-visit readings to out-of-office monitoring and ambulatory measurements, depending on clinical guidelines and physician recommendations.^2,3^ Regular BP monitoring in daily life is also recommended for early detection and effective healthcare management.^2,4^ However, current self-monitoring techniques, primarily cuff-based, can be uncomfortable and difficult for patients to maintain over time, especially for frequent or ambulatory measurements.^5,6^ This highlights the need for a more comfortable, user-friendly method for at-home hypertension screening.

Photoplethysmography (PPG), which measures peripheral blood volume changes during the cardiac cycle, is commonly used in wearable devices^7^ and has shown promise in cardiovascular monitoring, including arrhythmia detection and real-time BP estimation.^8,9^ Machine learning techniques have been applied to PPG-derived indices, including morphological, temporal, and derivative features, to assess cardiovascular status.^10,11^ However, PPG still faces several challenges: The signal is sensitive to motion artefacts and contact pressure, and its stability can be affected by variations in amplitude and waveform shape across devices, populations and environmental conditions.^12–14^ As a result, many previous studies have been limited to single datasets, restricting the generalizability of their findings across diverse populations and settings.^9,15–17^

Ambulatory BP monitoring is now recognized as a more accurate and physiologically relevant alternative to office-based measurements. It provides comprehensive readings, reduces the riskof white coat hypertension, and minimally disrupts daily activities.^2,18^ Crucially, it enables the detection of nocturnal BP patterns, which are key indicators of cardiovascular risk. Normotensive individuals typically exhibit a nocturnal ‘sleep-dip’ in BP, while hypertensive individuals may show non-dipping or reverse dipping patterns.^19^ Nighttime readings are often more reliable due to reduced motion and temperature fluctuations.^20^ Therefore, ambulatory 24-h PPG monitoring has the potential to capture awake–sleep variations in cardiovascular dynamics using a single wearable device,^21,22^ mapping intra-individual changes, and minimizing measurement errors associated with isolated readings, while highlighting outliers through continuous data collection.^18^ Despite its potential, PPG remains underutilized for hypertension risk screening, with most studies limited to short-term recordings or single-dataset models.^23–25^ Few have examined whether diurnal PPG variations reflect cardiovascular health status.

This study introduces a novel machine learning-based method for hypertension screening using ambulatory PPG measurements. The method is grounded on the hypothesis that normotensive individuals would exhibit greater awake–sleep differences in PPG features than those with unmanaged hypertension. This is because PPG reflects peripheral blood volume and its features can indicate peripheral arterial stiffness.^26^ In individuals with hypertension, arterial stiffness is often elevated and less variable, particularly at night,^27^ potentially reducing the circadian modulation of PPG-derived features. In contrast, normotensive individuals may retain greater vascular plasticity and thus exhibit more pronounced day–night variation. To test this hypothesis, we used two independent datasets: Aurora-BP (*n* = 180) for model development and internal validation, and CUHK-BP (*n* = 26) for external testing to assess generalizability.

## Methods

### Dataset description

**Figure 1:**
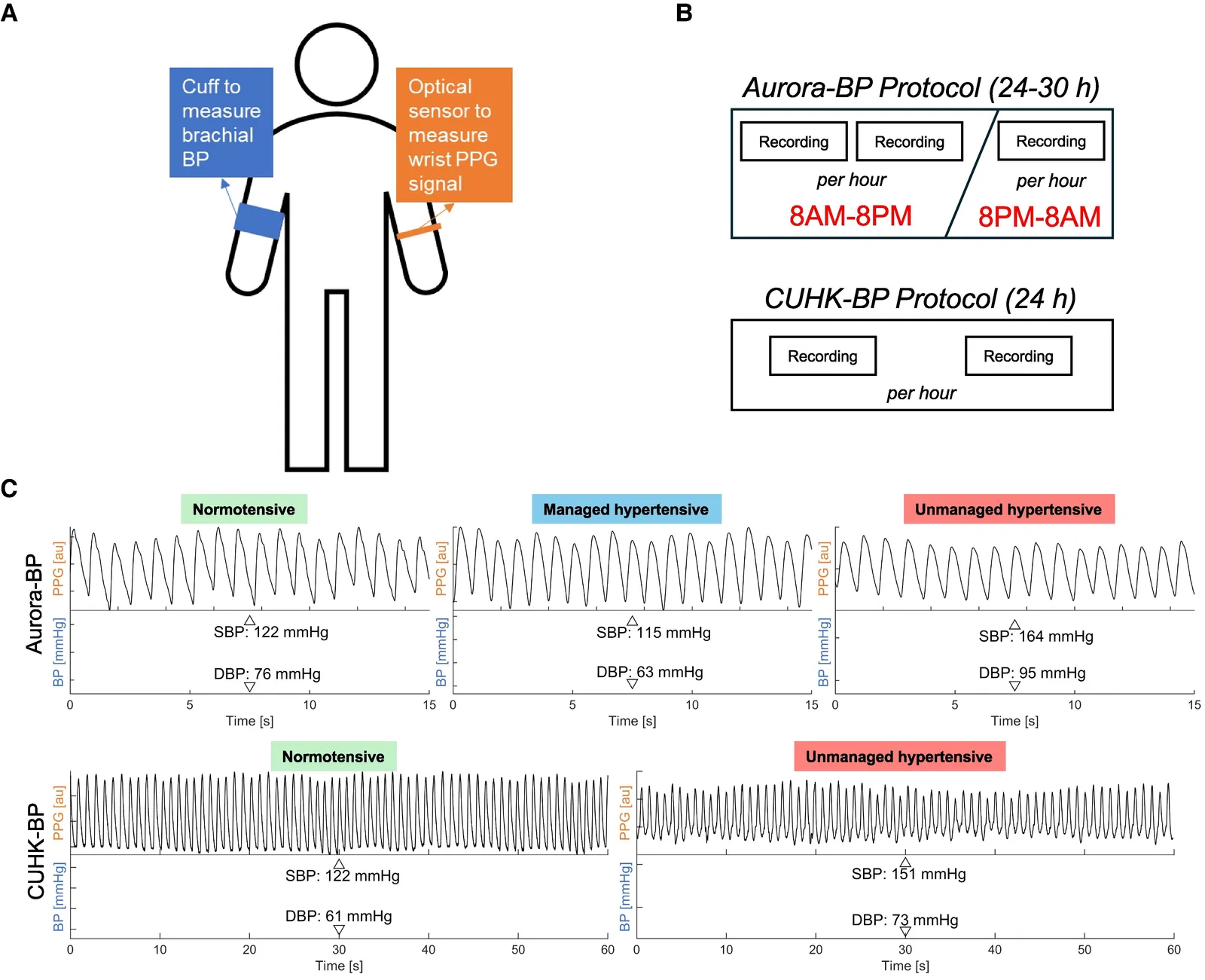
Ambulatory photoplethysmography (PPG) segments and corresponding blood pressure (BP) readings from the Aurora-BP and CUHK-BP datasets. (*A*) Data collection sites; (*B*) Measurement protocols: Aurora-BP, every 30 min during daytime (8 a.m. to 8 p.m.) and every 60 min during nighttime (8 p.m. to 8 a.m.); CUHK-BP, every 30 min over 24 h; (*C*) Example PPG segments with corresponding BP readings from representative normotensive, managed hypertensive, and unmanaged hypertensive subjects in the Aurora-BP dataset (top) and CUHK-BP dataset (bottom).

The Aurora-BP dataset is an open-access resource containing data from 1125 participants, collected to investigate cuff-based brachial BP readings, including systolic BP (SBP) and diastolic BP (DBP), along with Lead-I electrocardiogram (ECG), wrist PPG, and radial tonometric pressure waveforms.^28^ This study used only the data collected in the ambulatory setting (483 participants). PPG signals were recorded from the left wrist using an optical sensor (MAX30101, Analog Devices), while brachial BP was measured on the right arm using an ambulatory BP monitor (OnTrak 90227, Spacelabs Healthcare) (*Figure 1A*). Before ambulatory data collection, each participant was fitted with devices and rested in the lab to obtain initial brachial BP readings as a static baseline. During ambulatory monitoring, BP and PPG data were collected every 30 min during the daytime (8 a.m. to 8 p.m.) and every 60 min at night (8 p.m. to 8 a.m.), producing 15-s PPG segments paired with single SBP and DBP measurements (*Figure 1B* and *1C*).^28^

**Figure 2:**
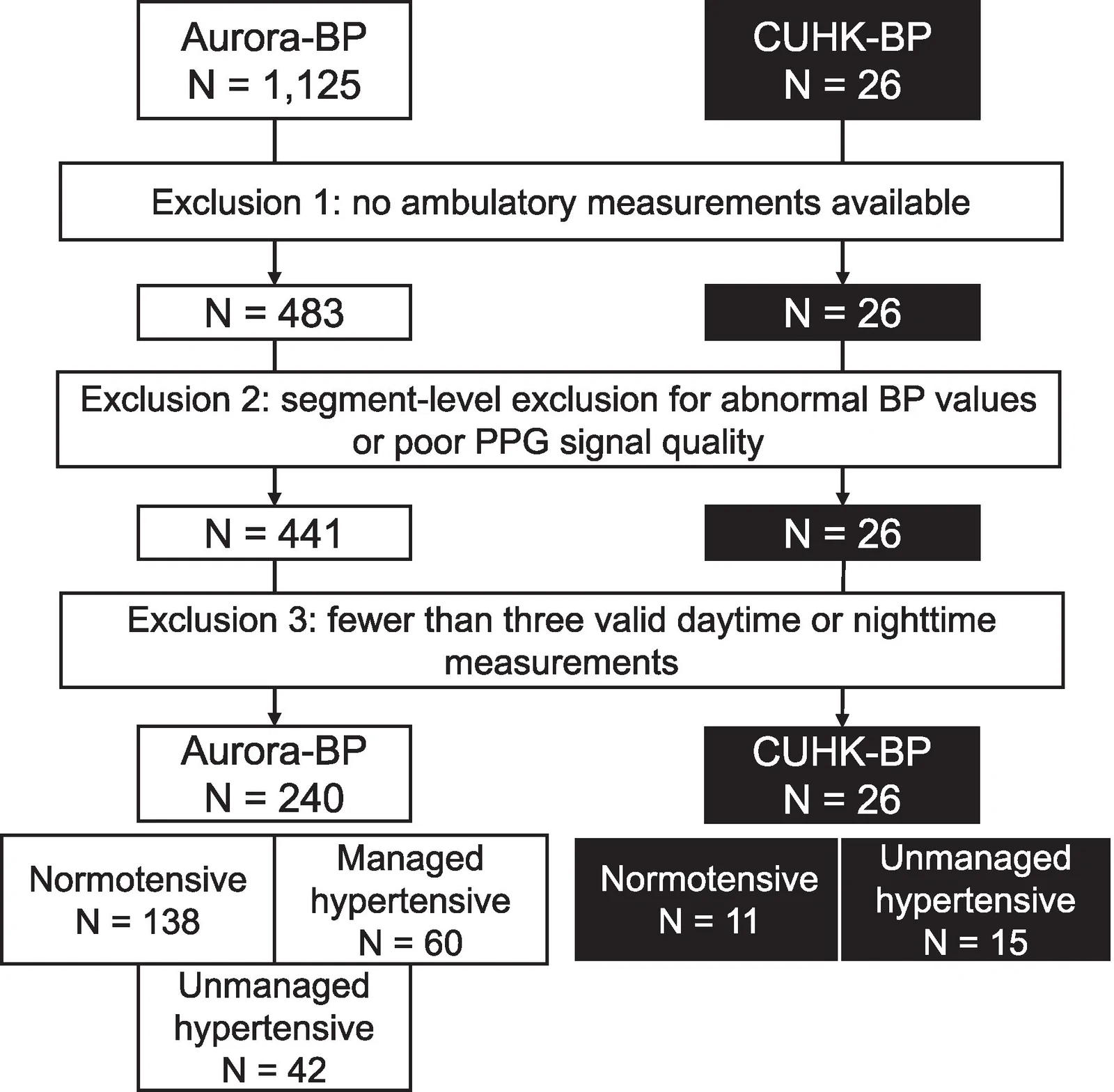
Cohort selection flow chart for the Aurora-BP (left) and CUHK-BP (right) datasets. Participants were excluded based on missing ambulatory data, abnormal blood pressure (BP) values, poor photoplethysmography (PPG) signal quality, or insufficient valid daytime or nighttime measurements.

Among the 483 participants with ambulatory recordings, segment-level preprocessing was applied to exclude recordings with abnormal BP values (DBP <30 mmHg)^29^ or poor-quality PPG signals. PPG signal quality was assessed using the heuristic score described in,^28^ which penalizes non-physiological artefacts (e.g. motion or electrical interference), low signal-to-noise ratio, and lack of cyclic consistency between consecutive pulses. A threshold of 0.8 was applied, below which reliable feature extraction was not feasible. After this step, 441 participants retained valid data. Participants with fewer than three valid daytime or nighttime measurements were then excluded to ensure that individual-level awake–sleep comparisons were based on repeated observations in both periods. This yielded a final Aurora-BP cohort of 240 participants (*Figure 2*).

Participants were classified as normotensive, managed hypertensive, or unmanaged hypertensive (*Table 1*). Hypertensive status was self-reported based on the 2017 AHA guidelines,^30^ and classification labels were prelabelled during the in-lab assessment. Participants with unmanaged hypertension were not receiving any antihypertensive treatment during the data collection period, while those with managed hypertension continued their usual treatment. This dataset did not provide detailed medical histories for all participants. Only the normotensive and unmanaged hypertensive participants were used for model training, testing, and evaluation as the internal dataset.

**Table 1:** Characteristics of Aurora-BP cohorts used in this study

	Normotensive	Managed hypertensive	Unmanaged hypertensive	*P*-value
No. of subjects	138	60	42	
PPG segments per subject	17 ± 7	16 ± 7	17 ± 8	0.739
PPG segment length, seconds	15	15	15	
Age, years	45 ± 9	49 ± 8	49 ± 9	0.002**
Female (%)	70 (51%)	22 (37%)	16 (38%)	0.115
Height, m	1.72 ± 0.10	1.73 ± 0.10	1.71 ± 0.10	0.632
Weight, kg	84.0 ± 21.5	95.3 ± 23.9	95.6 ± 28.3	0.001**
BMI, kg/m^2^	28.4 ± 6.9	31.8 ± 7.2	32.5 ± 8.9	1.2 × 10^−4^**
HR, bpm	77.2 ± 13.4	78.6 ± 15.3	80.2 ± 13.3	0.532
Static SBP, mmHg	120.9 ± 13.8	128.1 ± 13.1	139.0 ± 15.2	8.8 × 10^−11^**
Static DBP, mmHg	74.4 ± 8.5	77.8 ± 9.1	84.8 ± 10.5	2.9 × 10^−8^**
Sleep duration, hours	6.8 ± 2.3	6.9 ± 2.3	7.1 ± 2.4	0.766

Data are shown as value or mean ± SD for continuous variables and count (%) for categorical variables. Overall differences among the three groups were assessed using several statistical tests. Static BP readings refer to laboratory measurements taken prior to ambulatory data collection. Kruskal-Wallis tests were used to compare continuous variables among the normotensive, managed hypertensive, and unmanaged hypertensive cohorts, while chi-squared tests were used for categorical variables.

BMI, body mass index.

*0.01 ≤ *P* < 0.05; **P < 0.01.

The CUHK-BP dataset, collected by the Chinese University of Hong Kong and Prince of Wales Hospital,^21^ includes 24-h ambulatory data from 26 participants. Measurementes were obtained using a commercial BP monitor (Oscar2, SunTech Medical) and a custom armband equipped with a near-infrared PPG sensor and ECG electrodes (two e-textile and one Ag-Ag/Cl). PPG signals were recorded from the left upper arm, while BP was measured on the right arm (*Figure 1A*). Data were collected every 30 min over the 24-h period, yielding 60-s PPG segments paired with single SBP and DBP readings (*Figure 1B* and *1C*). Static BP measurements were also acquired in the lab prior to ambulatory monitoring as reference values. No participants were excluded (*Figure 2*). Demographic information is provided in *Table 2*. Participants were classified as normotensive or unmanaged hypertensive based on the 2010 Hong Kong Reference Framework^31^ and confirmed hospital diagnoses. As with the Aurora dataset, the CUHK dataset comprised participants with unmanaged hypertension who were not receiving antihypertensive treatment during the measurement period. Additionally, detailed medical histories were not available for all participants. This dataset was used exclusively for external model evaluation.

**Table 2:** Characteristics of CUHK-BP cohorts used in this study

	Normotensive	Unmanaged hypertensive	*P*-value
No. of subjects	11	15	
PPG segments per subject	33 ± 5	28 ± 6	0.022*
PPG segment length, seconds	60	60	
Age, years	26 ± 4	63 ± 4	2.0×10^−5^**
Female (%)	6 (54%)	10 (67%)	0.530
Height, m	1.71 ± 0.13	1.58 ± 0.08	0.025*
Weight, kg	63.5 ± 13.4	73.4 ± 8.2	0.697
BMI, kg/m^2^	21.4 ± 1.8	24.4 ± 2.6	0.005**
HR, bpm	74.5 ± 9.4	76.2 ± 10.3	0.716
Static SBP, mmHg	120.4 ± 11.6	137.9 ± 21.5	0.033*
Static DBP, mmHg	70.4 ± 6.9	73.4 ± 8.4	0.275
Sleep duration, hours	5.7 ± 1.8	6.4 ± 1.2	0.364

Data are shown as value or mean ± SD for continuous variables and count (%) for categorical variables. Differences between the two groups were assessed using several statistical tests. Static BP readings refer to laboratory measurements taken prior to ambulatory data collection. Mann–Whitney U-tests were used to compare continuous variables between the normotensive and unmanaged hypertensive cohorts, while chi-squared tests were used for categorical variables.

BMI, body mass index.

*0.01 ≤ *P* < 0.05; **P < 0.01.

### Signal processing

**Figure 3:**
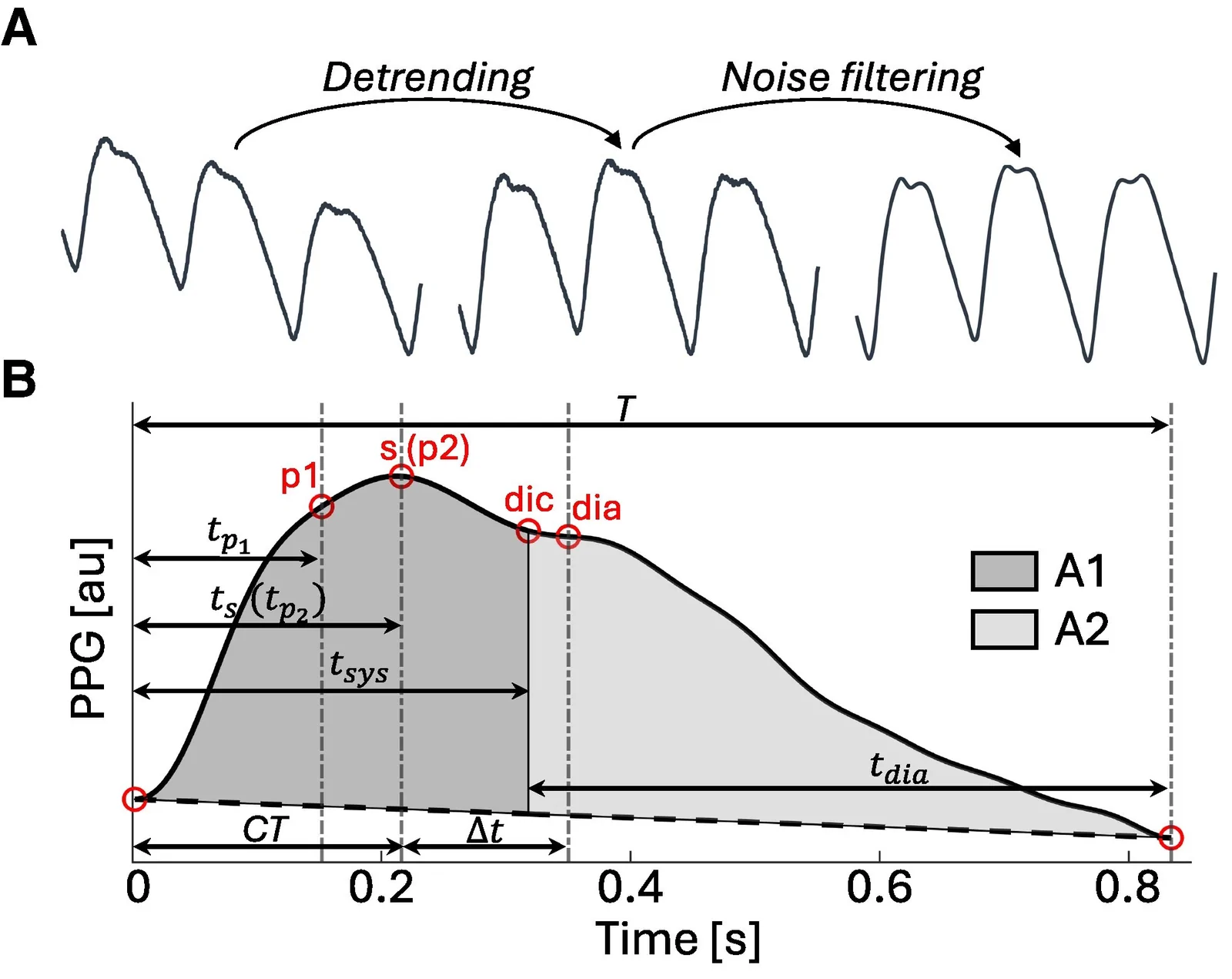
(*A*) Signal processing steps for PPG waveform pre-processing; (*B*) illustration of the PPG fiducial points used to compute the PPG-based indices listed in Supplementary material online, *Table S1*. All labels are defined in Supplementary material online, *Table S1*.

The 60-s PPG segments in the CUHK-BP dataset were trimmed to 25 s by extracting the middle portion to remove unstable waveforms and noise typically found at the segment boundaries. Raw PPG segments from both datasets were then filtered using a 4th-order high-pass Butterworth filter (cut-off: 0.25 Hz) to eliminate low-frequency noise and baseline wander, followed by an equiripple low-pass finite impulse filter with 10 Hz passband, 12 Hz stopband, 1 dB passband ripple, and 60 dB stopband attenuation to suppress high-frequency noise (*Figure 3A*). Fiducial points were extracted from each beat within the filtered PPG segments using the PulseAnalyse algorithm^32^ (*Figure 3B*). From these fiducials, 20 indices (11 temporal, 9 morphological) were computed as described in Supplementary material online, *Table S1*. Besides, heart rate (HR) was derived from the PPG signals by identifying systolic peaks and calculating the inter-beat intervals. The inverse of each interval yielded instantaneous HR. The average value of each index across all beats in a segment was then calculated for subsequent analysis.

### Awake and sleep data selection

In the Aurora-BP dataset, step counts from the 30 min preceding each PPG recording were used to define awake and sleep periods. Sleep onset was identified as the first occurrence of zero steps after 10 p.m., indicating sustained inactivity, while awake onset was defined as the first non-zero step count after sleep onset or after 6 a.m. These criteria delineated the awake and sleep periods (see Supplementary material online, *Figure S1*). In the CUHK-BP dataset, participant self-reported their sleep times, allowing direct separation of awake and sleep data. It is worth noting that these definitions were not based on clinical actigraphy. Instead, ‘awake’ refers to the daytime activity period, while ‘sleep’ refers to the nighttime rest period.

### Statistical analysis

Awake and sleep measurements were compared at both the cohort and individual levels. At the cohort level, paired *t*-tests were used to assess awake–sleep differences in SBP, DBP, mean BP (MBP = (SBP+2*DBP)/3),^33^ pulse pressure (PP = SBP-DBP), and HR across the three Aurora-BP cohorts and the two CUHK-BP cohorts. Differences between awake and sleep periods for all 20 PPG indices were also analysed using paired *t*-tests to assess statistical significance. Kruskal–Wallis tests were used to compare static SBP, DBP, MBP, PP, and continuous demographic variables among the three Aurora-BP cohorts, while Mann–Whitney *U*-tests were used for the same comparisons between the two CUHK-BP cohorts. Categorial variables were compared using chi-squared tests.

At the individual level, circadian SBP patterns were calculated as the percentage drop from mean awake to mean sleep SBP and classified as dipper (10–20% reduction), extreme dipper (≥20% reduction), non-dipper (<10% reduction), or reverse dipper (mean sleep SBP higher than mean awake SBP).^34^ Awake–sleep differences in the 20 PPG indices were evaluated separately for each subject in the normotensive and unmanaged hypertensive groups in both datasets. Due to the unequal numbers of awake and sleep measurements within individuals, Mann–Whitney *U*-tests were used. The resulting *P*-values reflected each index’s ability to differentiate awake and sleep states while remaining robust to outlier values (particularly important given the noise sensitivity of PPG signals). To normalize and simplify machine learning model inputs, *P*-values were discretized into three categories: (i) highly statistically significant (*P* < 0.01) was set to 1, (ii) statistically significant (0.01 ≤ *P* < 0.05) was set to 0.5, and (iii) not statistically significance (*P* ≥ 0.05) was set to zero. These 20 discretized *P*-values were used as input features for machine learning models. The frequency of significance labels was analysed across individuals within and between cohorts in the internal dataset to assess intra-cohort variability and inter-cohort separability.

### Machine learning models

Four binary classifiers, logistic regression (LR), K-nearest neighbours (KNN), support vector machines (SVM), and eXtreme Gradient Boosting (XGBoost), were used to classify subjects as normotensive or hypertensive. These models have previously been applied in hypertension screening studies.^23,35,36^

**Figure 4:**
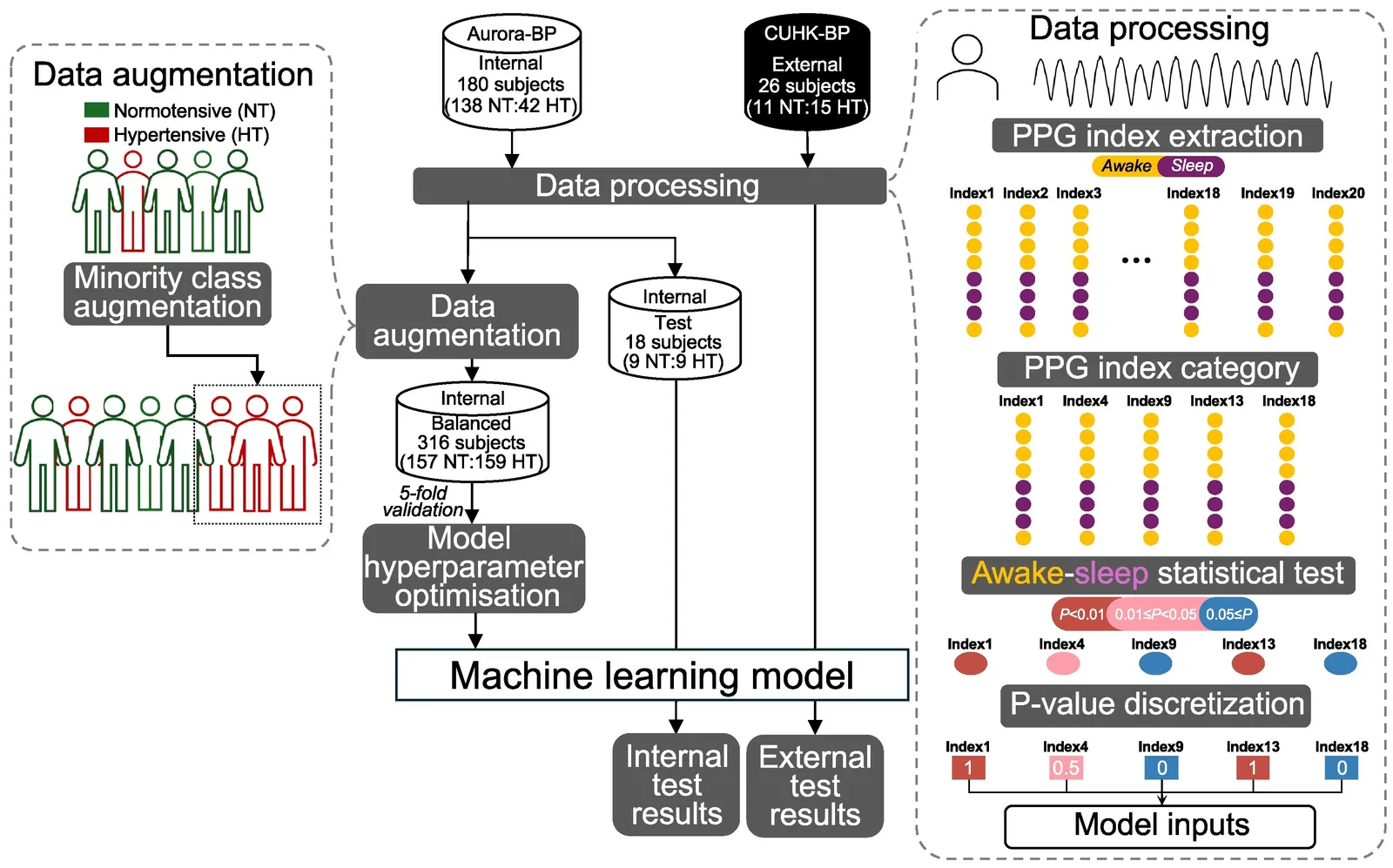
Flowchart of the model training and testing pipeline, including data preprocessing, feature extraction, class balancing, model training, hyperparameter optimization, and evaluation on internal and external datasets.

The same processing pipeline, illustrated in *Figure 4* was applied to each model. A randomly selected subset of 18 subjects (9 normotensive, 9 unmanaged hypertensive, 10% of Aurora-BP) was held out as an independent internal test set. The remaining 162 normotensive and unmanaged hypertensive subjects were used for training and validation. To address class imbalance (129 normotensive vs. 33 hypertensive), the Synthetic Minority Oversampling TEchnique (SMOTE)^37^ was applied using five nearest neighbours to synthetically balance the dataset (157 normotensive, 159 hypertensive). The internal test sets and the external CUHK-BP dataset were used for model evaluation.

All models were trained and optimized on discretized *P*-values from 20 PPG indices using the hyperparameters listed in Supplementary material online, *Table S2*. Five-fold cross-validation was applied during training. Model performance was evaluated using accuracy and F1-score on both internal and external test sets. Internal testing included five random splits to ensure robustness. For external testing, models were trained on the entire internal dataset. To assess the impact of longer PPG segments in the external dataset (60 s vs. 15 s), a sensitivity analysis was performed by dividing each 60 s segment into four non-overlapping 15 s segments, generating four external test subsets.

The 20 PPG-based indices were grouped into three categories: complete, temporal, and morphological. The complete group included all 20 indices listed in Supplementary material online, *Table S1*. The temporal group comprised 11 indices derived from time-fiducial points of the PPG waveform, while the morphological group included the remaining 9 indices extracted from morphological fiducial points.

The best-performing model was further analysed using SHapley Additive exPlanations (SHAP)^38^ to evaluate the contribution of each index to classification decisions.

### Baseline age-only model

Given that age is a well-established risk factor for hypertension,^39^ a baseline model was created using age as the sole input to the best-performing classifier. Due to the significant age difference between normotensive and unmanaged hypertensive subjects in the CUHK-BP dataset (*Table 2*), this model was trained and evaluated only on the internal Aurora-BP dataset to avoid confounding.

All signal processing, index extraction, and model development were performed in MATLAB (R2023a, MathWorks). A pseudocode representation of this workflow is shown in Supplementary material online, *Figure S2*.

## Results

### Awake and sleep differences

**Figure 5:**
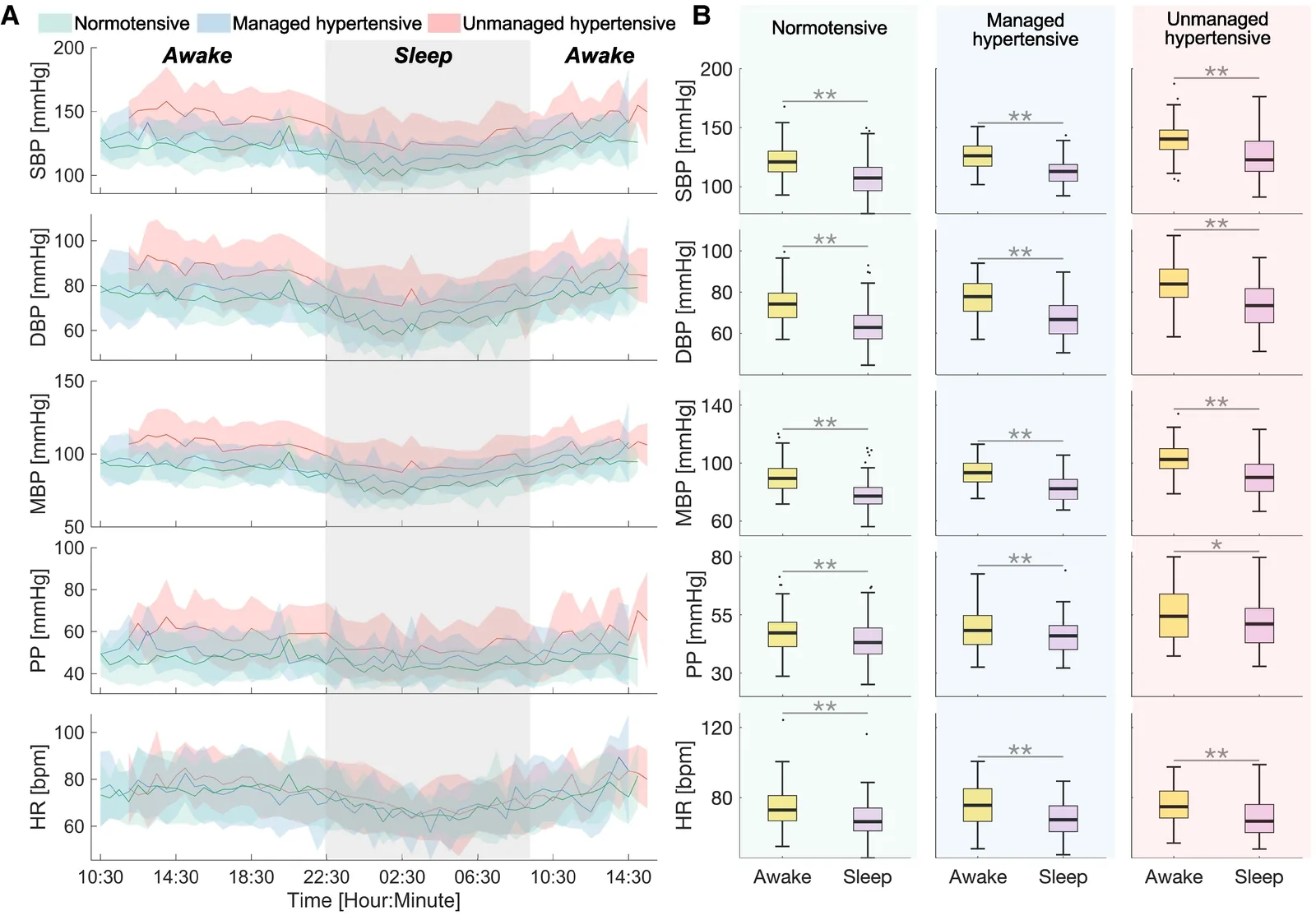
Blood pressure variations and statistical comparisons between awake and sleep periods across normotensive, managed hypertensive, and unmanaged hypertensive cohorts in the Aurora-BP dataset. (*A*) Trends in SBP, DBP, MBP, PP, and HR across the full 24-h ambulatory recording. Solid lines represent cohort means; shaded regions indicate standard deviations. (*B*) Statistical comparisons of SBP, DBP, MBP, PP, and HR between awake and sleep periods for each cohort. Paired t-tests were used to compare awake and sleep values within each cohort; *0.01 ≤ *P* < 0.05; **P < 0.01.

In the internal dataset, all three cohorts (normotensive, managed hypertensive, and unmanaged hypertensive) exhibited statistically significant reductions in SBP, DBP, MBP, PP and HR during sleep (*Figure 5B*). SBP, DBP, MBP, and HR followed similar circadian trends, with lowest values around 2:30 a.m. (*Figure 5A*). Although PP changes were less visually pronounced (*Figure 5A*), they remained statistically different (*Figure 5B*). Circadian SBP pattern distributions (dipper, extreme-dipper, non-dipper, and reverse dipper) were similar across the three cohorts (see Supplementary material online, *Table S3*). Significant differences in SBP, DBP, MBP, and PP were observed between normotensive and unmanaged hypertensive groups during both awake and sleep periods, while HR showed no significant difference between the two groups during either awake or sleep periods (see Supplementary material online, *Figure S3A*).

In the external dataset, DBP, MBP, and HR also decreased significantly during sleep, while SBP decreased only in the unmanaged hypertensive cohort (see Supplementary material online, *Figure S4*). PP showed no significant change. These trends were consistent with SBP pattern distributions (see Supplementary material online, *Table S3*), where the normotensive cohort had a high proportion of non-dippers (81.8%), while the unmanaged hypertensive cohort showed more dippers (33.3%) and reverse dippers (20.0%). All BP measures, except for SBP and PP during sleep, showed significant differences between normotensive and unmanaged hypertensive groups while HR showed no significant difference between the two groups during either awake or sleep periods (see Supplementary material online, *Figure S3B*).

**Figure 6:**
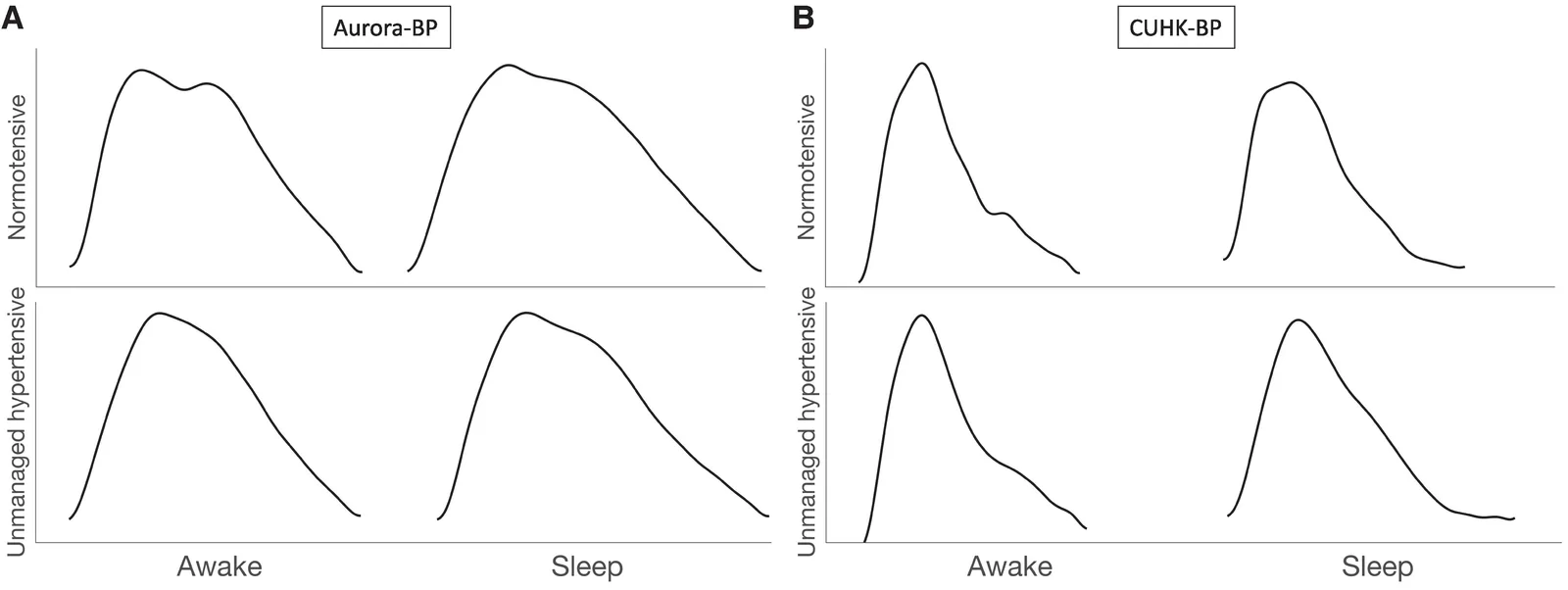
Example PPG waveforms recorded during awake and sleep periods in normotensive (top) and unmanaged hypertensive (bottom) individuals from the (*A*) Aurora-BP and (*B*) CUHK-BP datasets.

Representative PPG waveforms (*Figure 6*) were selected from randomly chosen subjects, with the displayed waveform segments selected by visual inspection to illustrate the typical awake–sleep differences observed in each group. In normotensive individuals, the awake–period waveform exhibited a clear dicrotic notch and diastolic peak, which were less prominent during sleep and in hypertensive subjects. Multiple PPG-derived indices also showed significant awake–sleep differences, particularly in the normotensive group (*Table 3*). Three temporal indices ($t_{sys}$, $t_{dia}$, $t_{\;p1-dia}$) and three morphological indices (*RI*, $radio_{\;p2-p1}$, and *A2*) were highly significant in normotensives (*P* < 0.01), but not in hypertensives (*P* ≥ 0.05). Four additional temporal indices (Δt, *SI*, $prop_{s}$, $t_{radio}$) and two morphological indices $(RI_{\;p2}$ and $ratio_{\;p2-p1}$) show moderate significance in normotensives (0.01 ≤ *P* < 0.05), but again were not significant in hypertensives. The managed hypertensive cohort showed a *P*-value distribution more closely aligned with the normotensive group, differing in only five indices.

**Table 3:** Statistical comparison of awake and sleep PPG indices across the three Aurora-BP cohorts

Index	Normotensive	Managed hypertensive	Unmanaged hypertensive
Δt	*	-	-
*CT*	**	**	**
*SI*	*	-	-
CT/h	**	**	**
$prop_{s}\;$	*	*	-
$t_{sys}$	**	**	-
$t_{dia}$	**	**	-
$t_{ratio}$	*	*	-
$prop_{\Delta t}$	**	**	*
$t_{\;p_{1}-dia}$	**	**	-
$t_{\;p_{2}-dia}$	-	-	-
*AI*	**	**	**
*AP*	**	**	**
*RI*	**	**	-
$RI_{\;p_{1}}$	**	*	*
$RI_{\;p_{2}}$	*	*	-
$ratio_{\;p_{2}-p_{1}}$	**	-	-
A1	**	**	*
A2	**	**	-
*IPA*	-	*	-

Paired t-tests were used to compare awake and sleep values within each cohort. A dash (-) indicates no statistical significance; * denotes statistical significance (0.01 ≤ *P* < 0.05); ** indicates high statistical significance (*P* < 0.01).

Although most indices were not statistically significant across all subjects (see Supplementary material online, *Figure S5*), normotensive individuals showed a higher proportion of significant indices than unmanaged hypertensives (8.3% vs. 3.2%). Notably, several indices (CT/h, $t_{sys}$, $t_{dia}$, $t_{\;p1-dia}$, *AI*, *AP*, $RI_{\;p1}$, $ratio_{\;p2-p1}$ and A1) were highly significant (*P* < 0.01) in over 10% of normotensive subjects.

### Hypertension screening performance

The SVM model achieved the strongest performance across both datasets, although using different index sets (*Table 4*). Internally, SVM attained the highest accuracy (81.1% ± 8.4%) and F1 score (82.8% ± 8.1%) using all 20 indices, whereas externally it achieved 84.6% accuracy and an 86.7% F1 score using only temporal indices. LR and XGBoost also performed well externally with temporal indices, each exceeding 70% accuracy and 80% F1 score. In contrast, KNN showed consistently poor performance, with internal scores below 63% and external scores below 45%. Overall, the complete index set was most effective internally, while temporal indices yielded better generalizability externally.

**Table 4:** Classification performance of the four models on internal and external test datasets using different categories of PPG-based indices

	Index category	Logistic Regression (LR)		K-Nearest Neighbours (KNN)		Support Vector Machines (SVM)		eXtreme Gradient Boosting (XGBoost)	
		Accuracy	F1-score	Accuracy	F1-score	Accuracy	F1-score	Accuracy	F1-score
Internal dataset	Complete	65.6% (4.7%)	71.1% (2.0%)	63.3% (5.0%)	61.2% (4.2%)	**81.1% (8.4%)**	**82.8% (8.1%)**	81.1% (6.3%)	82.1% (6.2%)
	Temporal	65.6% (6.1%)	69.0% (6.8%)	53.3% (3.0%)	29.6% (5.5%)	72.2% (8.8%)	73.8% (9.8%)	71.1% (4.7%)	71.2% (7.1%)
	Morphological	62.2% (8.2%)	70.0% (5.4%)	48.9% (8.2%)	23.3% (11.4%)	65.6% (8.2%)	69.2% (7.5%)	72.2% (11.8%)	77.6% (8.0%)
External dataset	Complete	69.2%	69.2%	42.3%	44.4%	57.7%	52.2%	61.5%	61.5%
	Temporal	73.1%	75.9%	50.0%	38.1%	**84.6%**	**86.7%**	80.8%	82.8%
	Morphological	73.1%	74.1%	38.5%	27.3%	61.5%	58.3%	61.5%	58.3%

Internal dataset accuracy and F1-score are reported as mean % (standard deviation %). External dataset accuracy and F1-score are reported as single values (%). Bold values indicate the highest accuracy or F1-score among the four models for each index category within each test dataset.

For the best-performing SVM model, internal testing showed a higher true negative rate (0.91) than true positive rate (0.71), with a false-negative rate of 0.29 and a false-positive rate of 0.09. External performance was more balanced across metrics (see Supplementary material online, *Figure S6*).

The internal and external test sets exhibited different circadian SBP pattern distributions compared with the training/validation set (see Supplementary material online, *Table S4*); however, no clear association with model performance was observed. Moreover, the best-performing model achieved a mean accuracy of 84.6% ± 3.1% across all external test subsets, indicating that differences in segment length between the internal and external datasets did not substantially affect model performance.

**Figure 7:**
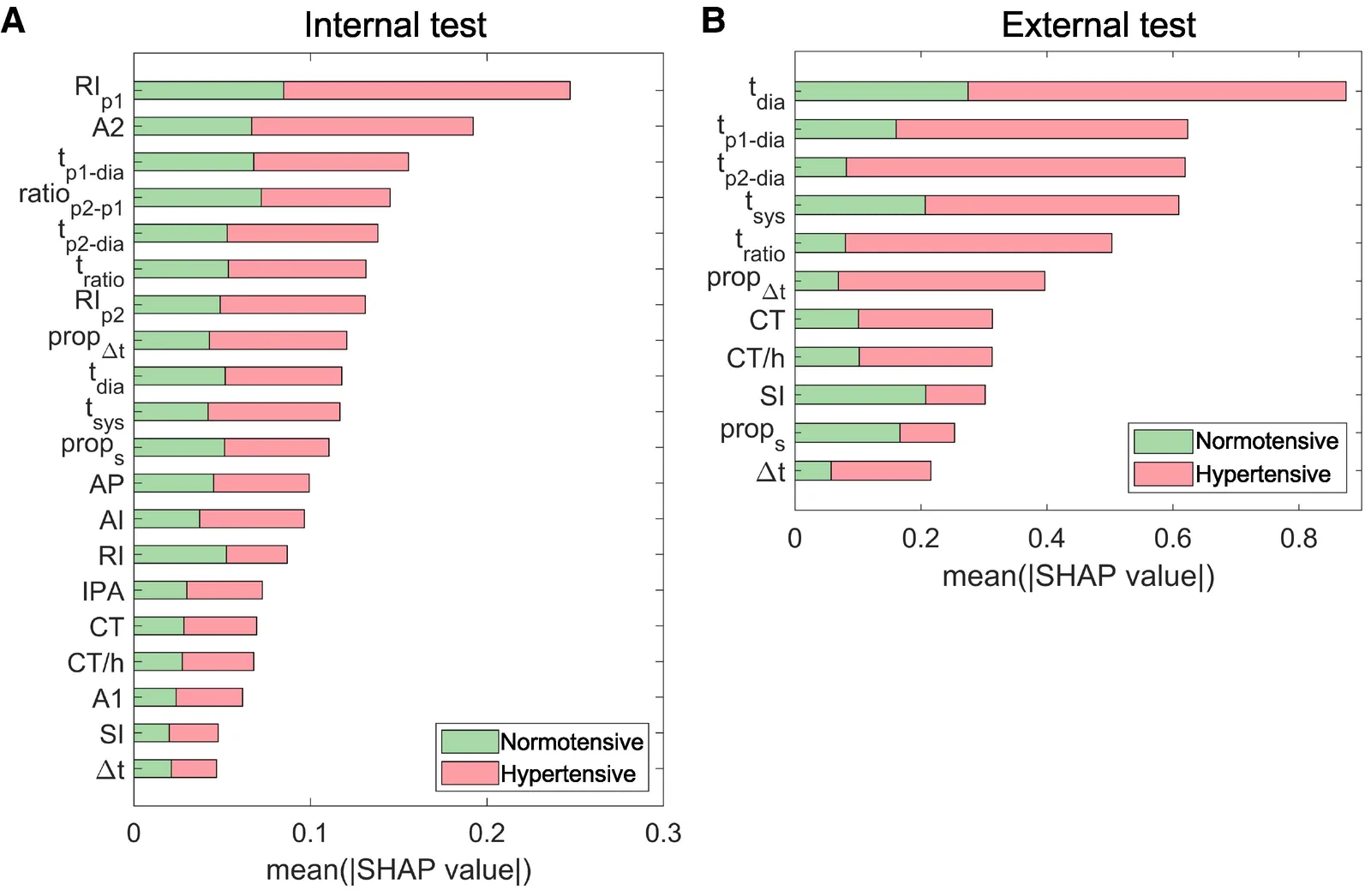
SHapley additive exPlanations (SHAP) values computed using the support vector machines model for (*A*) the complete set of PPG-based indices on the internal (Aurora-BP) test dataset and (*B*) the temporal indices on the external (CUHK-BP) test dataset. Green bars represent the mean absolute SHAP values contributing to normotensive classifications; red bars represent those contributing to hypertensive classifications.

SHAP analysis identified four morphological and six temporal indices among the top 10 most important features for hypertension classification in the internal test using all 20 indices (*Figure 7A*). In the external test, the most important indices were $t_{dia}$, $t_{\;p1-dia}$, $t_{\;p2-dia}$, $t_{sys}$, and $t_{ratio}$; all related to the dicrotic notch (*Figure 7B*).

The baseline SVM model using age alone achieved 67.8% ± 12.7% accuracy and a 70.5% ± 10.7% F1 score internally, indicating that age was a moderate but weaker predictor than PPG-derived indices.

Compared with previous studies, our *P*-value-based SVM approach achieved competitive accuracy and F1 scores on both internal and external datasets (*Table 5*), outperforming methods in^24^ and,^35^ and approaching those in,^23,25^ and,^40^ which lacked external validation.

**Table 5:** Comparison of the proposed method with previous studies to classify unmanaged hypertension from normotension

Input	Training	Test	Model	Accuracy	F1-score
17 PPG indices^24^	16 subjects (private dataset)	1 subject (private dataset)	AdaBoost		63%
10 PPG indices^23^	80 MIMIC subjects	80 MIMIC subjects	KNN		86.9%
4 PPG indices^35^	140 MIMIC III recordings	140 MIMIC III recordings	SVM	72.9%	77.0%
A PPG index^40^	30 subjects (private dataset)	13 subjects (private dataset)	Multiple instance learning	85.5%	89.2%
24 PPG indices^25^	405 Aurora-BP subjects	405 Aurora-BP subjects	Causality graphs + LR	89.0%	87.0%
20 complete PPG indices (this study)	162 Aurora-BP subjects	18 Aurora-BP subjects	Circadian statistical analysis + SVM	81.1% (8.4%)	82.8% (8.1%)
11 temporal PPG indices (this study)	180 Aurora-BP subjects	26 CUHK-BP subjects	Circadian statistical analysis + SVM	84.6%	86.7%

Each study is characterized by the input type (1st column), training dataset and sample size (2nd column), test dataset and sample size (3rd column), machine learning model used (4th column), accuracy (5th column), and F1-score (6th column).

## Discussion

This study demonstrated the feasibility of hypertension risk screening using ambulatory PPG measurements through a novel machine learning framework that leverages intra-subject awake–sleep differences in PPG morphology. The framework was guided by a physiological hypothesis that awake–sleep differences in PPG features differ between normotensive and hypertensive individuals and was evaluated within a 24-h ambulatory BP monitoring protocol, providing an interpretable basis for feature selection and model development. Grounding the model in physiological reasoning should strengthen its credibility among clinicians and physiologists, and to support future translation into clinical practice. Analysis of two independent datasets revealed distinct circadian variations in PPG-derived indices between normotensive and unmanaged hypertensive individuals, enabling effective classification. The support vector machine achieved the highest performance, with around 85% accuracy and F1-score on an external dataset unseen during training. Temporal indices proved particularly robust across datasets, highlighting their generalizability. The proposed *P*-value-based approach matched or outperformed previous methods and, importantly, included external validation; highlighting its potential for non-invasive, continuous hypertension screening in ambulatory settings.

Previous studies have shown that normotensive individuals typically exhibit a nocturnal BP dip, while hypertensive individuals may display altered circadian patterns.^19^ Findings from the Aurora-BP dataset were broadly consistent with this literature, with the proportion of dippers decreasing from 38.4% in normotensive individuals to 31% in unmanaged hypertensives. However, the CUHK-BP cohort was too small to support meaningful comparisons of dipping-pattern distributions. Despite these similar BP trends, 13 of the 20 PPG indices exhibited highly significant awake–sleep differences in the normotensive cohort, compared with only four in the unmanaged hypertensive group. This suggests that PPG captures distinct physiological responses to sleep not reflected by BP alone. The managed hypertensive cohort showed PPG patterns more closely aligned with the normotensive group, indicating that treatment may partially restore normal cardiovascular function.^41^ Notably, although BP-based sleep–awake differences were less pronounced in the external dataset, PPG-based indices still effectively distinguished normotensive from unmanaged hypertensive individuals, demonstrating the sensitivity of our novel *P*-value-based approach to subtle sleep-related cardiovascular changes.

At the individual level, both normotensive and hypertensive subjects exhibited many non-significant awake–sleep differences. However, normotensives individuals had a higher frequency of highly significant changes, reflecting greater physiological variability and supporting the use of machine learning to leverage these differences for classification.

Temporal indices yielded the strongest external performance, suggesting they capture hypertension-related features that generalize better than morphological indices, which may be more dataset-specific. These findings are consistent with previous work demonstrating the robustness of temporal features across PPG sensors and anatomical sites.^11^

Unlike some previous work that included demographic features such as age,^42^ we excluded demographic features from the final PPG-based model, as normotensive and hypertensive cohorts in the external dataset differed significantly in age (*Tables 1* and *2*). This allowed a focused assessment of PPG-derived features, while the age-only baseline model was used to assess the potential contribution of age. Although age alone was a moderate predictor in our internal dataset, the PPG-based model substantially outperformed the age-only baseline model on the internal dataset. Other demographic characteristics (height, weight, and body mass index [BMI]) also differed between groups. In the internal dataset, weight was greater in the hypertensive cohorts (*Table 1*), whereas in the external dataset, height was higher in normotensives (*Table 2*). Both datasets exhibited significantly higher BMI in unmanaged hypertensives. These differences highlight the heterogeneity between datasets and demonstrate that our model captures intra-subject PPG variations independent of demographic factors.

Among the models tested, SVM consistently outperformed others across datasets. KNN performed moderately on the internal dataset but poorly on the external one, likely due to its reliance on distance-based similarity metrics, which are sensitive to data distribution shifts. In contrast, SVM, XGBoost, and LR, which learn underlying patterns, showed stronger cross-dataset robustness. SVM’s advantage may stem from its focus on critical samples near the decision boundary rather than fitting the entire dataset to find optimal decision boundaries,^43^ making it more robust in small, imbalanced datasets. Indeed, the internal dataset’s imbalance led SVM to favour normotensive predictions internally, while using temporal indices externally produced more balanced classification outcomes.

SHAP analysis identified the most influential features for classification. In the internal dataset, $RI_{\;p1}$ and *A2* (both morphological indices) ranked highest, consistent with their established association with arterial stiffness and vascular ageing; factors implicated in disrupted diurnal BP variation in hypertension.^44^ Temporal indices were more influential in the external dataset. Previous ICU-based work using 24-h measurements has shown that circadian rhythms in $t_{sys}$, $t_{ratio}$, and *CT* correlate more strongly with SBP than with heart rate, while Δt shows no correlation.^45^ Our findings aligned with these observations, except that *CT* demonstrated moderate importance in both datasets. Notably, the top five ranked indices ($t_{dia}$, $t_{\;p1-dia}$, $t_{\;p2-dia}$, $t_{sys}$, and $t_{ratio}$) are all related to the dicrotic notch of the PPG waveform, which marks aortic valve closure in the cardiac cycle. During sleep, heart rate typically decreases and valve closure is delayed in normotensives, a pattern often disrupted in hypertension.^46–48^ These disruptions may explain the high importance and strong generalizability of dicrotic-notch-related indices in our SHAP analysis.

To assess whether HR variations drive the performance of PPG-based hypertension models, we examined wake–sleep HR changes across both datasets. All cohorts showed similar nocturnal HR dipping, regardless of hypertension status (*Figure 4* and Supplementary material online, *Figure S4*), suggesting that HR alone does not explain the models’ classification performance. However, as temporal PPG features may still partly reflect HR-related effects, the contribution of HR to model performance should be evaluated more directly in future work.

This study has several limitations. First, both datasets were relatively small, particularly the external dataset, so performance estimates may be affected by sampling variability. In addition, the requirement of at least three valid daytime and nighttime measurements is a relatively low threshold and may limit the robustness of individual-level comparisons, although restricting the analysis to validated measurements helped ensure that retained data reflected genuine pulsatile physiology rather than artefacts. Furthermore, differences in ambulatory acquisition intervals and PPG segment durations between datasets may have influenced the estimation of awake–sleep variability and feature stability. Larger and more diverse cohorts are needed to further validate generalizability, although the inclusion of external validation provides an important initial assessment of model transferability. Second, we performed binary classification, whereas clinical guidelines define multiple hypertension stages.^2,3,30^ Third, we excluded managed hypertensive subjects from classification due to their absence in the external dataset. Fourth, ‘awake’ and ‘sleep’ periods were approximated based on activity or self-reports rather than clinical actigraphy, which may introduce bias.

## Conclusions

This study introduced a novel machine learning-based method for hypertension screening that leverages intra-subject awake–sleep differences in PPG indices. This approach effectively distinguished normotensive from hypertensive individuals and demonstrated strong generalizability on an independent dataset. Temporal indices, particularly those related to the dicrotic notch, proved more robust across datasets than morphological indices. To our knowledge, this is the first study to exploit awake–sleep PPG variations for hypertension assessment, highlighting the potential of wearable devices for non-invasive, ambulatory monitoring in real-world settings.

## Supplementary Material

ztag131_Supplementary_Data

## Data Availability

The full Aurora-BP dataset underlying this article is available upon application through https://doi.org/10.5281/zenodo.19099166. The processed CUHK-BP data are publicly available at https://github.com/jingyuanhong/hypertension-screening-awake-sleep.
