# Supplementary material for: Hypertension screening via awake–sleep differences in photoplethysmogram signals
Source: Eur Heart J Digit Health. 2026 Aug 13;7(7):ztag131. doi: 10.1093/ehjdh/ztag131 (PMC13520938; doi:10.1093/ehjdh/ztag131)
Supplement: Supplementary file 1 [file ztag131_supplementary_data.pdf]

Online Supplement

Hypertension Screening via Awake-Sleep Differences in Photoplethysmogram Signals

Jingyuan Hong[1], Manasi Nandi[2], Yali Zheng[3], Jordi Alastruey[1]

[1] School of Imaging Sciences and Biomedical Engineering, King's College London, London, United Kingdom

[2] School of Cancer and Pharmaceutical Science, King's College London, London, United Kingdom

[3] College of Health Science and Environmental Engineering, Shenzhen Technology University, Shenzhen, China

Correspondence:

Dr Jordi Alastruey, School of Imaging Sciences and Biomedical Engineering, King's College London, St. Thomas' Hospital, London SE1 7EU, United Kingdom

Tel: + 44 020 7848 9556, E-mail: jordi.alastruey-arimon@kcl.ac.uk

Table S1: The 20 PPG-based indices used in this study.

Type	Index	Formula	Description
Temporal	Δt	$t_{dia} - t_s$	t_{dia}: time of diastolic peak; t_s: time of systolic peak
	CT	t_s	t_s: time of systolic peak
	SI	$h/\Delta t$	h: height of subject; Δt: time interval between time of diastolic peak and time of systolic peak
	CT/h	t_s/h	t_s: time of systolic peak; h: height of subject
	$prop_s$	t_s/T	t_s: time of systolic peak; T: time of pulse end
	t_{sys}	t_{dic}	t_{dic}: time of dicrotic notch
	t_{dia}	$T - t_{dic}$	T: time of pulse end; t_{dic}: time of dicrotic notch
	t_{ratio}	t_s/t_{dic}	t_s: time of systolic peak; t_{dic}: time of dicrotic notch
	$prop_{\Delta t}$	$(t_{dia} - t_s)/T$	t_{dia}: time interval between dicrotic notch and the end; t_s: time of systolic peak; T: time of cardiac cycle
	t_{p_1-dia}	$t_{dia} - t_{p_1}$	t_{dia}: time interval between dicrotic notch and the end; t_{p_1}: time of the early systolic peak
	t_{p_2-dia}	$t_{dia} - t_{p_2}$	t_{dia}: time interval between dicrotic notch and pulse end; t_{p_2}: time of the late systolic peak
Morphological	AI	$(x_{p_2} - x_{p_1})/x_s$	x_s: amplitude of systolic peak; x_{p_1}: amplitude of the early systolic peak; x_{p_2}: amplitude of the late systolic peak
	AP	$x_{p_2} - x_{p_1}$	x_{p_1}: amplitude of the early systolic peak; x_{p_2}: amplitude of the late systolic peak
	RI	x_{dia}/x_s	x_{dia}: amplitude of diastolic peak; x_s: amplitude of systolic peak
	RI_{p_1}	x_{dia}/x_{p_1}	x_{dia}: amplitude of diastolic peak; x_{p_1}: amplitude of the early systolic peak
	RI_{p_2}	x_{dia}/x_{p_2}	x_{dia}: amplitude of diastolic peak; x_{p_2}: amplitude of the late systolic peak
	$ratio_{p_2-p_1}$	x_{p_2}/x_{p_1}	x_{p_1}: amplitude of the early systolic peak; x_{p_2}: amplitude of the late systolic peak
	$A1$	$\int_{t_0}^{t_{dic}} x$	area from pulse foot to dicrotic notch
	$A2$	$\int_{t_{dic}}^{T} x$	area from dicrotic notch to pulse end
	IPA	$A2/A1$	A1: area from pulse foot to dicrotic notch; A2: area from dicrotic notch to pulse end

For each index (2nd column), the table provides the corresponding mathematical formula (3rd column), a description of the fiducial points used in the formula (4th column; illustrated in Figure 2b), and the index type (temporal or morphological, 1st column). x represents the PPG signal.

Table S2: Hyperparameters used for each machine learning model when tested on the external dataset. Values are shown for three feature sets: complete indices, temporal indices, and morphological indices.

	Complete indices	Temporal indices	Morphological indices
	LR		
Regularisation parameter	0.038	0.002	0.785
	KNN		
Number of nearest neighbours	3	1	3
	SVM		
Box constraint level	2048	2048	8192
Kernel scale parameter	1	1	2
	XGBoost		
Maximum number of splits	20	10	5
Minimum number of leaf nodes	20	20	10
Number of weak learners	100	200	10
Learning rate	1	0.5	1

Table S3: Proportions of circadian systolic blood pressure patterns (dipper, extreme dipper, non-dipper, and reverse dipper) in normotensive, managed hypertensive, and unmanaged hypertensive cohorts from the Aurora-BP dataset, and in normotensive and unmanaged hypertensive cohorts from the CUHK-BP dataset. Values in each row sum to 100%.

	Dipper (%)	Extreme-dipper (%)	Non-dipper (%)	Reverse dipper (%)
		Aurora-BP dataset		
Normotensive	38.4	14.5	37.7	9.4
Managed hypertensive	40.0	10.0	38.3	11.7
Unmanaged hypertensive	31.0	16.7	38.1	14.3
		CUHK-BP dataset		
Normotensive	9.1	0.0	81.8	9.1
Unmanaged hypertensive	33.3	0.0	46.7	20.0

Table S4: Proportions of circadian systolic blood pressure patterns (dipper, extreme dipper, non-dipper, and reverse dipper) in the training/validation and testing sets.

	Dipper (%)	Extreme-dipper (%)	Non-dipper (%)	Reverse dipper (%)
Aurora-BP dataset – training/validation				
Normotensive	39.4±0.7	13.8±1.2	37.8±0.7	9.0±1.2
Unmanaged hypertensive	24.4±9.3	24.4±16.5	35.6±9.3	15.6±16.9
Total	38.5±0.8	13.7±1.1	38.0±0.7	9.8±0.9
Aurora-BP dataset – testing				
Normotensive	35.2±3.5	13.3±3.5	38.8±2.5	12.7±4.5
Unmanaged hypertensive	15.6±12.7	28.9±12.7	35.6±9.3	20.0±16.5
Total	20.0±7.5	26.7±10.7	35.6±6.3	17.8±8.2
CUHK-BP dataset – testing				
Normotensive	9.1	0.0	81.8	9.1
Unmanaged hypertensive	33.3	0.0	46.7	20.0
Total	23.1	0.0	61.5	15.4

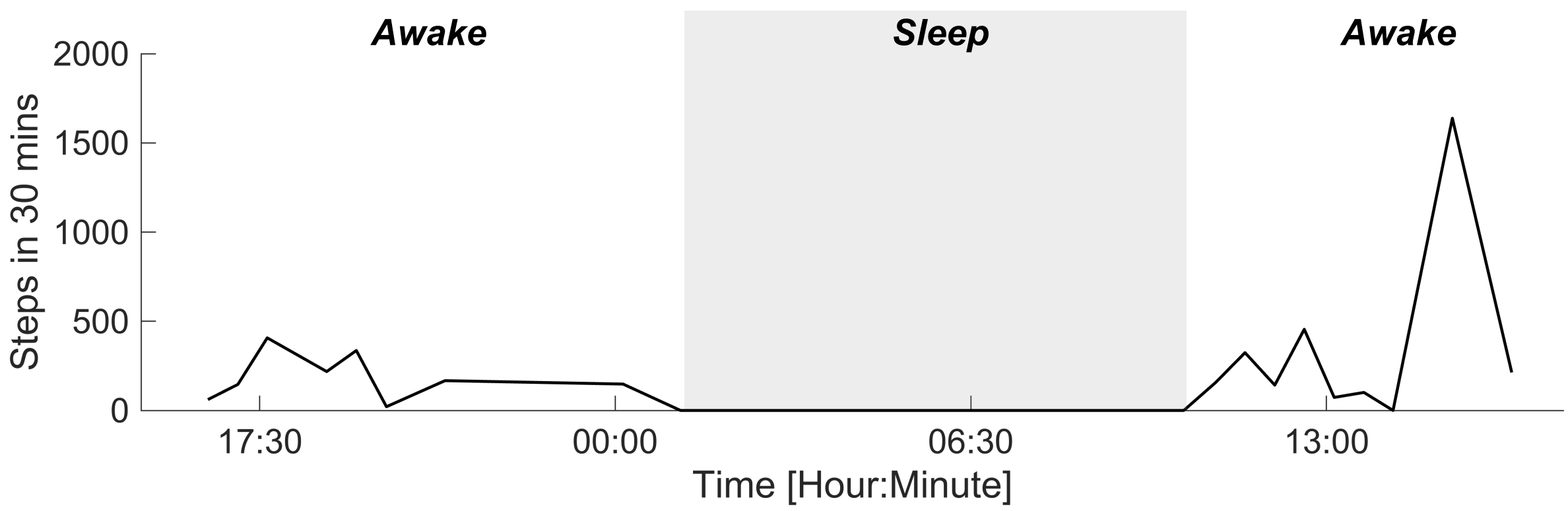

Figure S1: Example of 30-minute step-count data from a participant in the Aurora-BP dataset, illustrating the identification of awake and sleep onsets during 24-hour ambulatory monitoring.

Algorithm to build a machine learning model for hypertensive classification.

Input: $f_{i,n,status}$: $PPG\ indices\ matrix, where$
i: $number\ of\ PPG\ indices, n$: $number\ of\ subjects, status \in \{awake, sleep\}$;
L_n: $Labels\ for\ n\ subjects$;
θ: $hyperparameters$; rs: $random\ seed$; cv: $number\ of\ cross\ validation\ folds$

Output: $bestModel$, $bestScore$

1 **for** _in range n **do**
    for _in range i **do**
        $p = MannWhitneyUTest(f_{i,n,awake}, f_{i,n,sleep})$
    end
end
2 $p_{selected} = IndexSelectionStrategy(p)$
3 $X_{balanced}, Y_{balanced} = SMOTE(p_{selected}, L_n, rs)$
4 $Models = \{LR, KNN, SVM, XGBoost\}$, $bestScore = -\infty$, $bestModel = null$
5 **for** $model \in Models$ **do**
    for _in range *cv* **do**
        $X_{training}, Y_{training}, X_{validation}, Y_{validation} = split(X_{balanced}, Y_{balanced})$
        $bestParams = GridSearch(model, X_{training}, Y_{training}, \theta, rs)$
        $trainedModel = model(X_{training}, Y_{training}, bestParams)$
        $scoreValidate(cv) = trainedModel(X_{validation}, Y_{validation})$
        if $scoreValidate(cv) > bestScore$
            $bestScore = scoreValidate(cv)$
            $bestModel\{model\} = trainedModel$
        end
    end
end
6 **return** $bestModel, bestScore$

Figure S2: Pseudocode representation of the algorithm used to develop each machine learning model for hypertension classification.

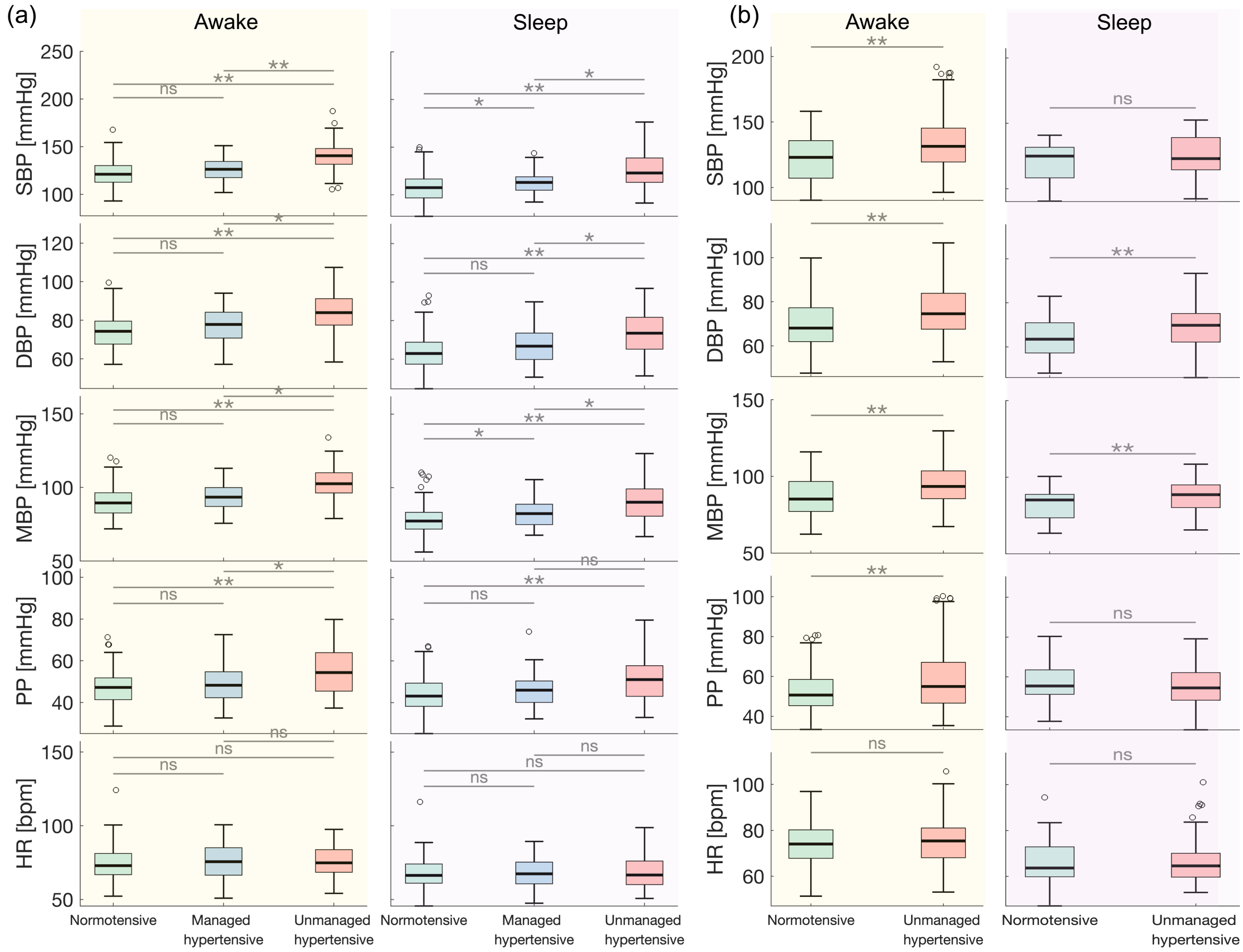

Figure S3: Statistical comparisons of SBP, DBP, MBP, PP, and HR among cohorts during awake and sleep periods in the (a) Aurora-BP dataset and (b) CUHK-BP dataset. Kruskal–Wallis tests were used for comparisons among the three Aurora-BP cohorts, whereas Mann–Whitney U-tests were used for comparisons between the two CUHK-BP cohorts; ns, not significant; *0.01 ≤ p < 0.05; **p < 0.01.

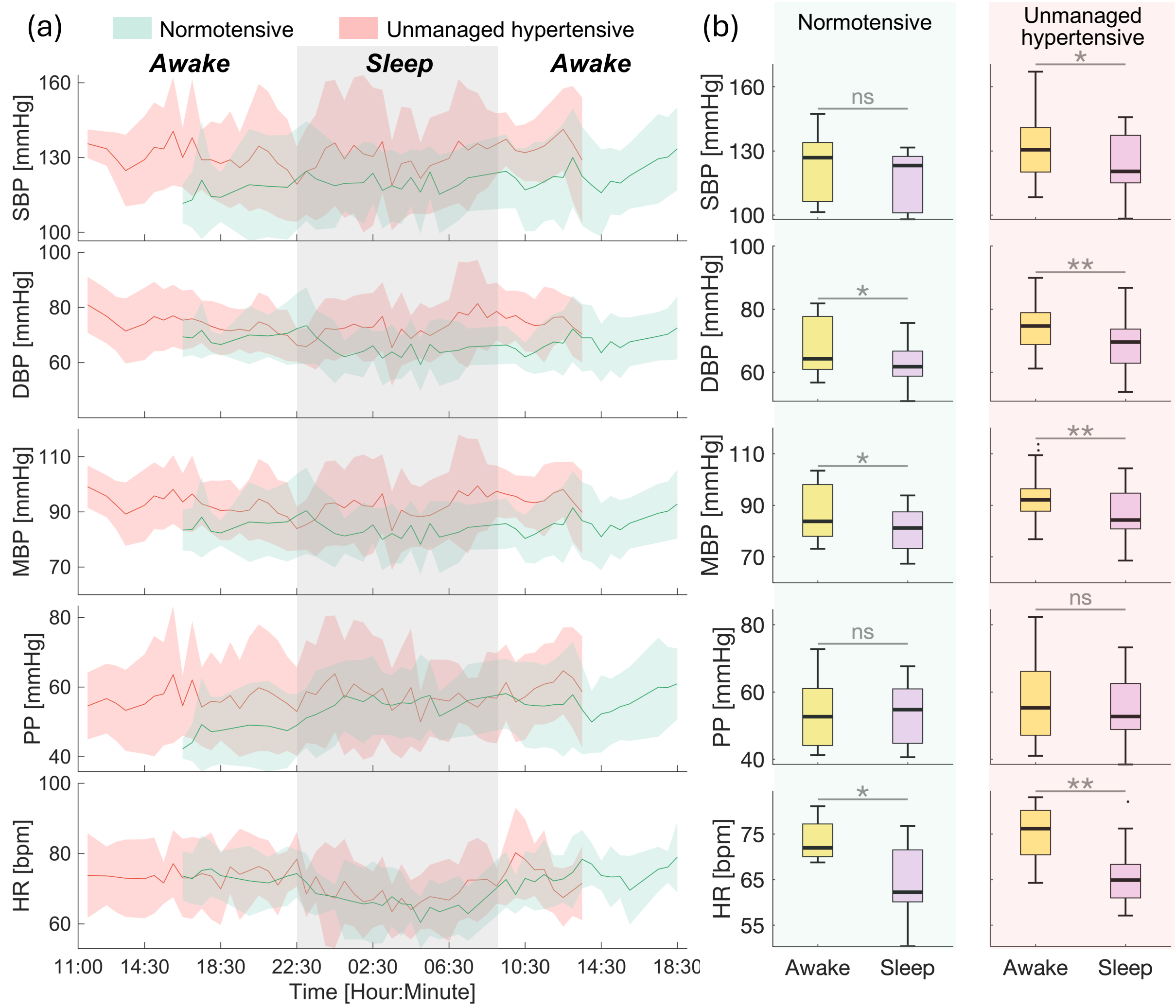

Figure S4: Blood pressure and heart rate (HR) variations and statistical comparisons between awake and sleep periods across normotensive and unmanaged hypertensive cohorts in the CUHK dataset. (a) Trends in SBP, DBP, MBP, PP, and HR across the full 24-hour ambulatory recording. Solid lines represent cohort means; shaded regions indicate standard deviations. (b) Statistical comparisons of SBP, DBP, MBP, PP, and HR between awake and sleep periods for each cohort. Paired t-tests were used to compare awake and sleep values within each cohort; ns, not significant; $*0.01 \leq p < 0.05$; $**p < 0.01$.

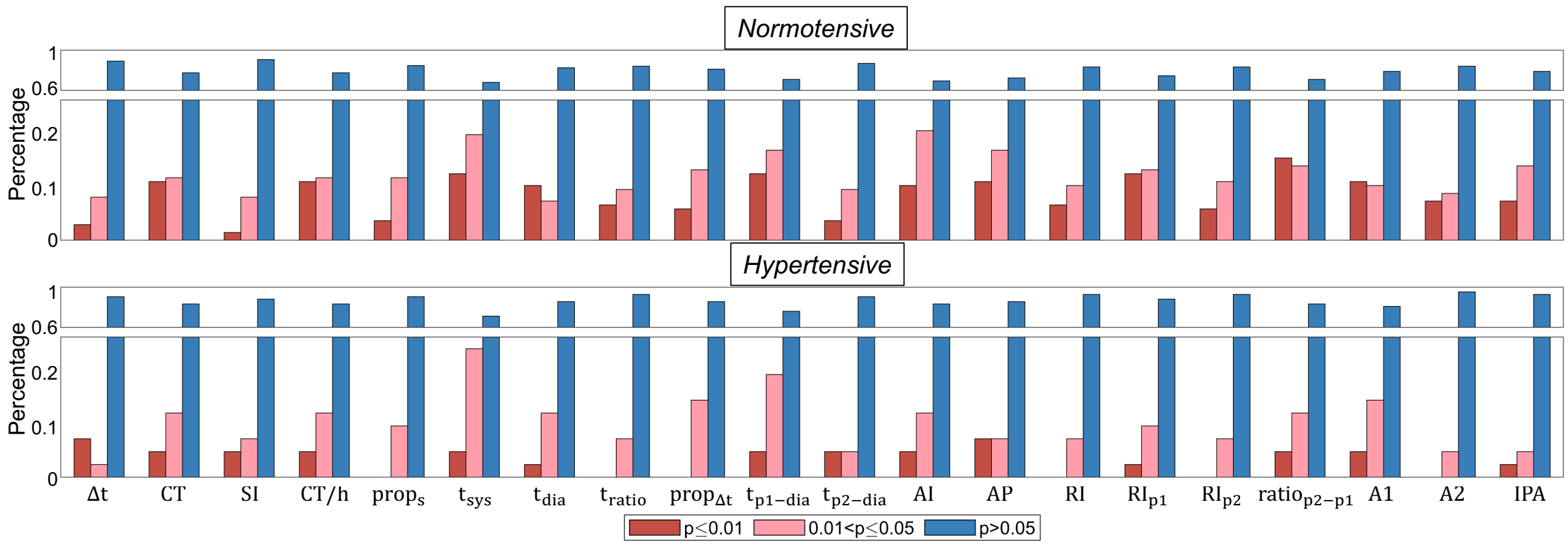

Figure S5: Frequency distribution of p-value ranges for all 20 PPG-based indices in normotensive (top) and unmanaged hypertensive (bottom) individuals from the internal Aurora-BP dataset. Highly significant values ($p < 0.01$) are shown in red, significant values ($0.01 \leq p < 0.05$) in pink, and non-significant values ($p \geq 0.05$) in blue. Mann-Whitney U-tests were used.

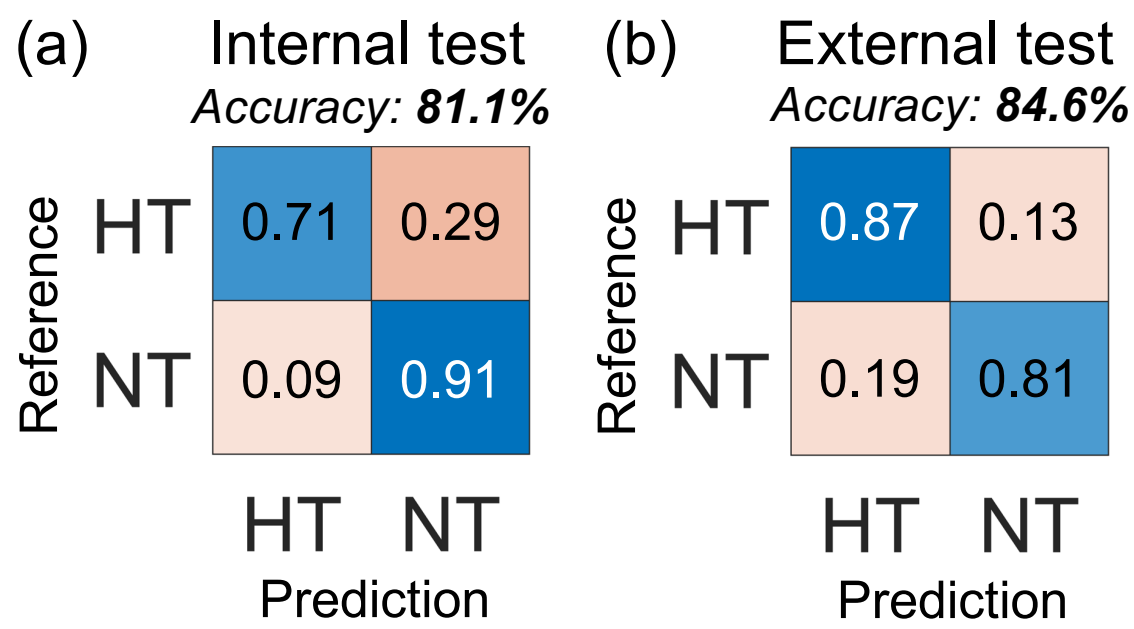

Figure S6: Best classification results of the Support Vector Machines model on (a) the internal Aurora-BP test dataset and (b) the external CUHK-BP test dataset. HT: hypertensive; NT: normotensive.
